# Preparing for the upcoming 2022/23 influenza season: A modelling study of the susceptible population in Australia, France, Germany, Italy, Spain and the United Kingdom

**DOI:** 10.1111/irv.13091

**Published:** 2022-12-28

**Authors:** Bronke Boudewijns, John Paget, Marco Del Riccio, Laurent Coudeville, Pascal Crépey

**Affiliations:** ^1^ Netherlands Institute for Health Services Research (Nivel) Utrecht The Netherlands; ^2^ Department of Health Sciences University of Florence Florence Italy; ^3^ Sanofi, Modeling Epidemiology and Data Science Lyon France; ^4^ EHESP, CNRS, Inserm, Arènes ‐ UMR 6051, RSMS – U 1309 Université de Rennes Rennes France

**Keywords:** Australia, COVID‐19, Europe, Influenza, modelling, susceptibility

## Abstract

We analysed the influenza epidemic that occurred in Australia during the 2022 winter using an age‐structured dynamic transmission model, which accounts for past epidemics to estimate the population susceptibility to an influenza infection. We applied the same model to five European countries. Our analysis suggests Europe might experience an early and moderately large influenza epidemic. Also, differences may arise between countries, with Germany and Spain experiencing larger epidemics, than France, Italy and the United Kingdom, especially in children.

## 1 INTRODUCTION

Before the COVID‐19 pandemic, influenza was responsible for 15 000–70 000 deaths and up to 50 million symptomatic cases every year in the European Union (EU) and European Economic Association (EEA).^1^ After April 2020 and during the 2020/21 winter, influenza circulation was massively reduced by the emergence of SARS‐CoV‐2 and the related non‐pharmaceutical interventions (NPIs).^2^ NPIs have been largely lifted in the EU/EEA and influenza co‐circulated with SARS‐CoV‐2 during the 2021/22 winter,^3^ and it is expected to make a stronger return during the 2022/23 winter.^4^ A number of news reports have looked at the influenza season in Australia and predicted alarming scenarios for the Northern Hemisphere.^5^

## 2 METHODS

We adapted an age‐structured SEIR‐like transmission model, which accounts for the impact of past influenza epidemics on the susceptibility for influenza in the population.^6^ The model was calibrated with laboratory‐confirmed detections^7^, ^8^ and burden estimates^9^, ^10^ to replicate the dynamic of strain specific influenza epidemics over the period 2012–2022 in Australia and in the five largest countries in Western Europe: France, Germany, Italy, Spain and the United Kingdom. Age‐stratified contact matrices in this model were based on the Mistry et al. model.^11^ Our model accounts for influenza vaccination and effectiveness (i.e., some vaccinated may still be susceptible), and this allows an estimation of the level of immunity acquired after an influenza epidemic and its progressive exponential waning over time (average duration of 6 years for influenza A). In all six countries, the vaccination coverage rates (VCRs) for influenza were collected and included in the model.^12^, ^13^ The United Kingdom was the only country that reached the WHO VCR target of 75% for people aged 65 years and older, with 81% in 2020/21 and 82% in 2021/22. Spain was the second country closest to meet the target with 69% in 2021/22. We also introduced the impact of NPIs on influenza transmission by using the outcomes of a COVID‐19 model^14^ in which country‐specific levels of NPIs were estimated as part of model calibration to reported cases, hospitalizations and deaths.^14^, ^15^

## 3 RESULTS

**FIGURE 1:**
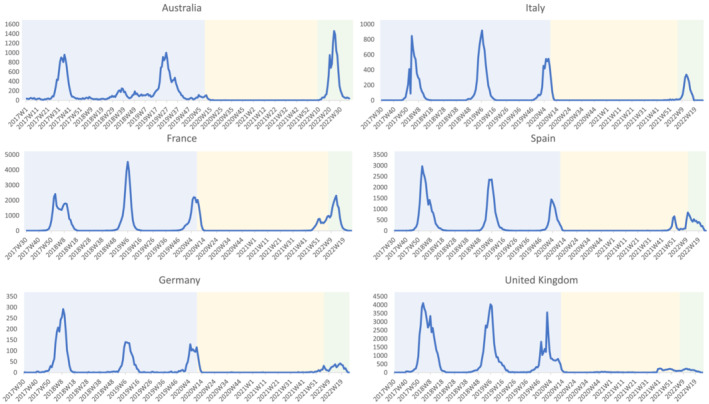
Weekly number of laboratory confirmed influenza detections, reported to FluNet, since the 2017 season in Australia and 2017/18 season in France, Germany, Italy, Spain and the United Kingdom. The period preceding the COVID‐19 pandemic is shown with a blue background, the period with most COVID‐19 related non‐pharmaceutical interventions (NPIs) and few influenza cases is shown with a yellow background, the period in 2022 after most NPIs were lifted per country^28^, ^29^ is shown with a green background.

Figure 1 displays the number of laboratory‐confirmed influenza detections per week from 2017–2022, available through FluNet.^8^ In the Southern Hemisphere, the influenza season in Australia usually starts in May and lasts until October.^16^ Following the emergence of SARS‐CoV‐2 and the implementation of strong NPIs, including heavy restrictions on travel to and from Australia, there was very little influenza activity until the winter of 2022. After a 2‐year absence, this season was characterised by an early appearance (mid‐April 2022) and a relatively high peak in influenza detections in June, followed by a steep decrease and the season was dominated by influenza A (mostly A(H3N2), if subtyped).^17^

The influenza season in Europe (Northern Hemisphere) typically lasts from November to April, with the number of cases usually peaking around February.^18^ In 2020/21, there were hardly any influenza detections (see Figure 1) in France, Germany, Italy, Spain and the United Kingdom, and the following winter of 2021/22 was generally mild, with a late influenza season dominated by influenza A(H3N2). There was some variation in influenza activity between countries; in the United Kingdom, the season started relatively early, but the peak was postponed in all five countries. Spain was the only country with two distinct peaks, and the United Kingdom had the lowest levels of laboratory detections compared with previous seasons.

**FIGURE 2:**
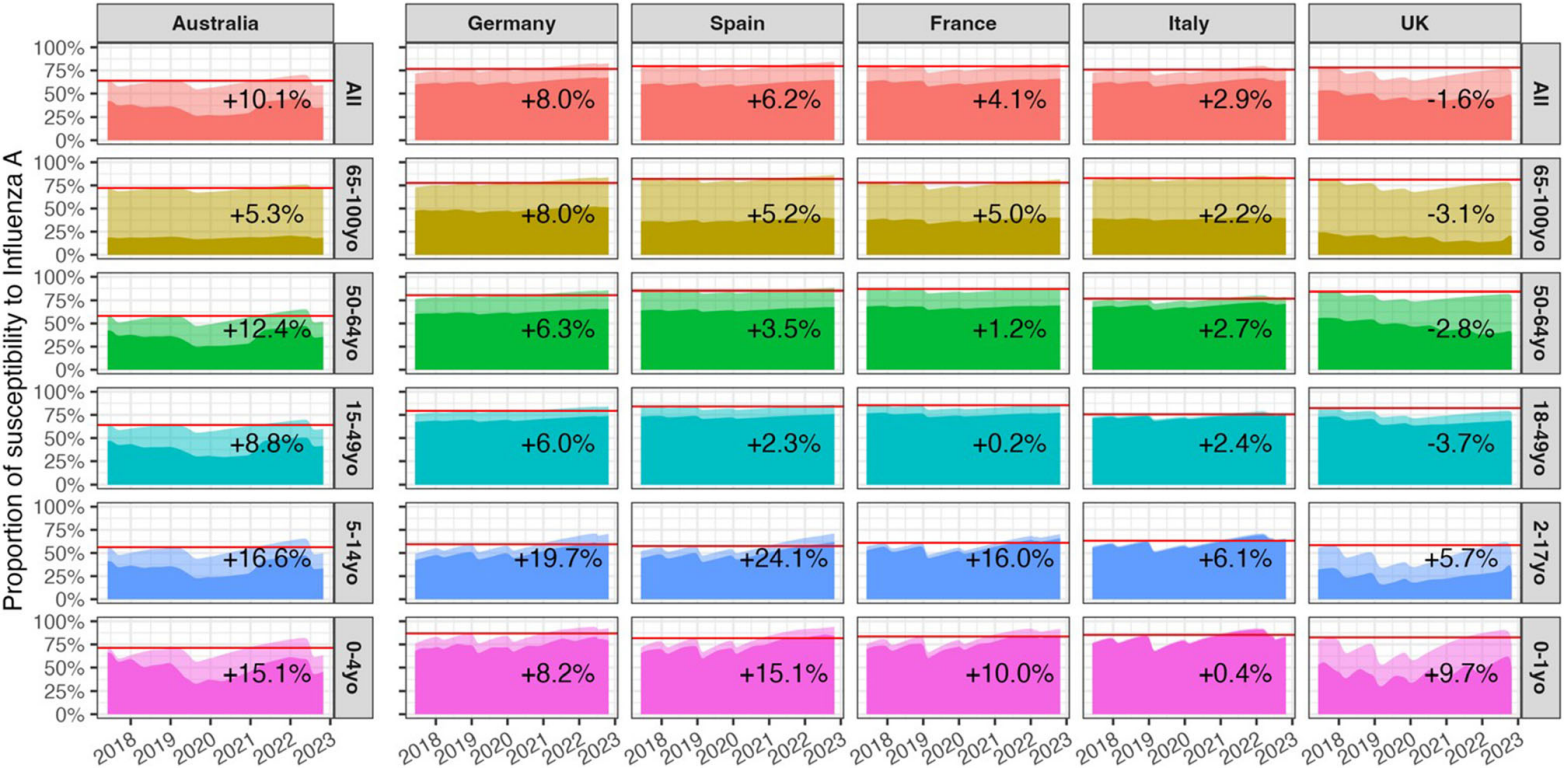
Evolution of the population susceptibility to influenza A (H1N1 and H3N2) since 2017 by age group in Australia, Germany, Spain, France, Italy and the United Kingdom. Horizontal red lines show the maximum level of susceptibility before January 2020. The percentage of increase (or decrease) displayed on the plots are computed for the highest susceptibility level ahead of the 2022 (Australia) and 2022–2023 (Europe) influenza season compared with the red lines. Transparent areas stand for susceptible who vaccinated.

Figure 2 displays the proportion of susceptible persons per country and age‐group from 2017 to 2022 for influenza A. Because vaccinated individuals remain partially susceptible, both vaccinated and unvaccinated persons are presented. Results for Australia showed an overall increase in the proportion of susceptible persons (+10.1%), ahead of the 2022 influenza season compared with the reference (maximum susceptible population between 2017 and January 2020). The estimated susceptible population was also higher in Germany (+8.0%) and Spain (+6.2%), and lower in the United Kingdom (−1.6%). The percentage increase compared with the reference was highest in the youngest two age‐groups in both Australia (0–4 and 5–14 years) and the five European countries (0–1 and 2–17 years). The susceptibility picture in Germany and Spain looked most like the one in Australia; in France and Italy, the proportion of susceptible persons did not increase as much. In the United Kingdom, the estimated susceptible population only exceeded the reference in the two youngest age‐groups.

## 4 DISCUSSION

**FIGURE 3:**
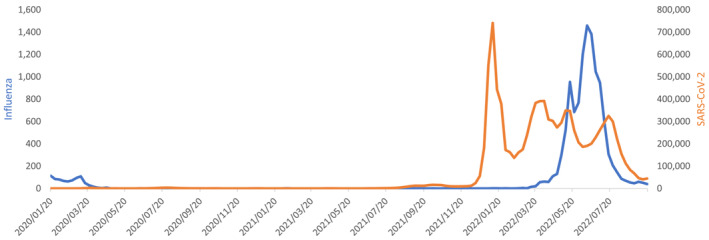
Influenza and SARS‐CoV‐2 detections in Australia, between January 2020 and September 2022

The 2022 influenza season in Australia was characterised by an early onset and a slightly shorter and sharper epidemic than previous seasons (see Figure 1). The number of laboratory‐confirmed detections was very high compared with previous seasons; however, primary care data showed low rates and the hospitalisation rate was average.^17^ Factors that could have influenced the duration of the influenza season were the rise of a new SARS‐CoV‐2 Omicron variant (see Figure 3) and the vaccination levels that are relatively high compared with Europe (68.3% in persons aged 65 years and over). Vaccination against influenza is recommended for anyone over 6‐months‐old and is available free of charge for certain risk groups.^19^ In addition, in June and July of 2022, some states (Queensland and Western Australia) provided the vaccine free of charge to all inhabitants over 6 months to increase vaccine uptake.^20^, ^21^ One difference between Australia and Europe is that Australia had no real influenza activity since the start of COVID‐19 pandemic, but Europe experienced some mild influenza activity during the 2021/22 winter, and this will affect the proportion of the population that is susceptible to influenza due to infections.

Predictive modelling can be an important tool to generate possible influenza activity scenarios. In these models, a crucial role is given to the level of susceptibility for influenza in the population, which is altered by both infection and vaccination. Our approach allows one to reconstruct the immune status of the population based on past epidemics. We then used the susceptible population metric to interpret influenza epidemics that occurred in Australia and to assess what may be expected this winter in Europe. Based exclusively on this metric, a larger than usual influenza outbreak could be expected in the five European countries. However, given that the largest overall increase in the susceptible population was seen in Australia and this did not lead to an extreme caseload, or increased number of hospitalisations due to influenza in 2022, a scenario with a very large outbreak in Europe seems unlikely. In addition, the highest levels of susceptibility increase are found in the young population (0–14 years for Australia and 0–17 years for the five European countries), a population where influenza circulation could be high, but probabilities of severe outcomes remain generally low. However, another study found that a 10–60% increase in the population susceptibility might lead to a maximum of onefold to fivefold rise in peak magnitude and a onefold to fourfold rise in the epidemic size for the upcoming 2022–2023 influenza season.^22^

An early rise in influenza detections is possible, especially in countries with the highest increase in the susceptible population. Germany has already reported an increase in influenza detections in late November 2022, just like the United States, Canada and Mexico.^23^ A similar early start was observed in Australia and also described in Chile,^24^ where the season was characterised by an early start and atypical course, but fewer hospitalisations, compared with before the COVID‐19 pandemic. An important factor that will impact the upcoming influenza epidemics in Europe will be the specific viruses that circulate in Europe. Some countries in the southern hemisphere had an H3N2 dominant season, such as Australia, Chile^24^ and Argentina,^25^ while others had an H1N1 dominant season (South Africa). The H1N1 epidemic in South Africa, was then followed by a separate influenza B epidemic.^26^ Another important factor would be the emergence of a new SARS‐CoV‐2 variant, which could impact influenza activity (see Figure 3 for how this played out in Australia), for example, via the implementation of NPIs.

One limitation of our study is that we have focussed our analysis on influenza A. This was a methodological choice as (a) influenza A is typically the dominant virus circulating in the population (B generally represents only 23% of detections in a season),^27^ (b) influenza B did not play an important role in influenza epidemics in Australia and the other southern hemisphere countries, and (c) our estimation of susceptibility is mainly illustrative and should only be used for relative comparisons between countries. Another limitation is that these are modelled estimates of susceptibility, which would be important to compare to serological data. Finally, our analysis was limited by some of the data inputs (e.g., the VCR data for Germany was limited).

In summary, the data from Australia suggest that Europe might experience an earlier epidemic than usual, but based on our estimates of the susceptible population and the hospitalisation rates in Australia and Chile, we would not expect a major influenza outbreak this winter in Europe. There may, however, be differences in the epidemics across Europe, with Germany and Spain experiencing larger epidemics, especially in children, compared with France, Italy and the United Kingdom. These results highlight the importance of monitoring and modelling the level of immunity against influenza across countries. They also depend on the European population maintaining their prevention and control measures, including influenza vaccination.

## CONFLICT OF INTEREST

BB and MDR report no conflicts of interest, PC reports consulting fees from Sanofi non‐related to this project, LC is an employee of Sanofi and JP reports that Nivel has received funding for influenza research projects from Sanofi, the Fondation de France and WHO.

## ETHICS STATEMENT

Ethical approval was not required for this study as all data used within this work were part of routine surveillance.

## AUTHOR CONTRIBUTIONS

**Bronke Boudewijns:** Investigation; writing‐original draft; writing‐review and editing. **John Paget:** Conceptualization; investigation; writing‐original draft; writing‐review and editing. **Marco Del Riccio:** Investigation; writing‐review and editing. **Laurent Coudeville:** Data curation; formal analysis; investigation; methodology; writing‐review and editing. **Pascal Crépey:** Conceptualization; formal analysis; investigation; methodology; writing‐original draft; writing‐review and editing.

### PEER REVIEW

The peer review history for this article is available at https://publons.com/publon/10.1111/irv.13091.

## Data Availability

Not applicable.
